# YTHDF2 inhibit the tumorigenicity of endometrial cancer via downregulating the expression of IRS1 methylated with m^6^A

**DOI:** 10.7150/jca.54527

**Published:** 2021-05-05

**Authors:** Ling Hong, Xiaowen Pu, Haili Gan, lichun Weng, Qingliang Zheng

**Affiliations:** Shanghai First Maternity and Infant Hospital, Tongji University School of Medicine, Shanghai 201204, China.

**Keywords:** YTHDF2, IRS1, endometrial cancer, AKT signaling pathway, m^6^A modification

## Abstract

RNA epigenetic modification take part in many biology processes, and the N6-methyladenosine (m^6^A) methylation of specific mRNAs in endometrial cancer (EC) tissues play a key role in regulating the tumorigenicity of EC, but the specific mechanism still unknown and need to be investigated in the future. Here, we found that m^6^A reader protein YTHDF2 expression was significantly upregulated in EC compare to tumor adjacent tissues, YTHDF2 was then identified to inhibit the proliferation and invasion of EC cell lines. Mechanistically, the m^6^A reader YTHDF2 bind the methylation sites of target transcripts IRS1 and promoted IRS1 mRNA degradation, consequently inhibiting the expression of IRS1 and inhibiting IRS1/AKT signaling pathway, finally inhibit the tumorigenicity of EC. Thus, we demonstrated that YTHDF2 inhibited the proliferation and invasion of EC via inhibiting IRS1 expression in m^6^A epigenetic way, which suggests a potential therapeutic target for EC.

## Introduction

Endometrial cancer (EC) is the most common gynecologic malignancy in the world 1. The majority of EC patients with abnormal vaginal bleeding diagnosed at early stage and cured by surgery resulted in 5-year survival rate of 95%, while other EC patients with atypical symptoms diagnosed with advanced or recurrent disease have a poor prognosis with 5-year survival rate of 17%, which seriously affect the health of women 2. Current common tests for EC diagnosis include ultrasound, hysteroscopy, pelvic examination, endometrial biopsy, dilation, and curettage 3. Moreover, 10-15% of EC patients with early stage will undergo a recurrence within 3 years after therapy 4. However, the available treatment options after initial therapy at the time of disease progression or recurrence are still limited 5. Therefore, early detection and follow-up are pivotal to heighten the survival rate of EC patients, meanwhile better understanding the molecular mechanism of EC and developing new therapies based on molecular signatures are needed to improve patient's survival rate, but the key mechanisms mediating tumorigenicity of endometrial cells are still not fully clear.

*N^6^*-methyladenosine (m^6^A) is the most prevalent mRNA modification in eukaryotes 6-8. The biological functions of this reversible modification are reported to participate in regulating RNA structure 9, translation 10 and degradation 11, the underlying mechanisms that mediate this dynamic regulation requires investigation. It has been reported that the dysregulation of m^6^A is involved in affecting cancer initiation and progression in a variety of cancers, such as hepatocellular carcinoma 12, EC 13, and renal cancer 14. Consistent with these roles, many investigations have been conducted to determine the relationship between m^6^A and cancer treatment 15, 16, suggesting that m^6^A regulation proteins can serve as therapeutic targets for cancers and as promising biomarkers for overcoming resistance 17. FTO overexpression promotes significant chemoresistance in cervical squamous cell carcinoma 18*.* Chen *et al* developed a novel prognostic risk signature based on three m^6^A-regulators (FTO, YTHDC1 and WTAP) 19. Recently reported that reduced m^6^A mRNA methylation as an oncogenic mechanism in EC and identify m^6^A methylation as a regulator of AKT signaling 8. However, whether and how the key target genes of YTHDF2 affect endometrial cell activity and the underlying signaling pathways mediate these changes are still far from elucidated.

Insulin Receptor Substrate (IRS) proteins play a central role in the cascade of insulin signaling 20. Upon insulin binding its cognate receptor, insulin signaling begins to transmit and causes the insulin receptor (IR) autophosphorylation and activation, which subsequently phosphorylates several scaffold molecules including the IRS family of proteins 21. To date, four members of this family of scaffold molecule have been identified in mammalian cells (IRS 1-4). Insulin signaling deficiency inhibited the proliferation and metastasis of cancer cells 22. It is reported that IRS1 plays a key role in cancer cell proliferation and mediates the resistance to anticancer drugs, while IRS2 acts mainly in cancer cell motility and metastasis 23. Phosphorylation of IRS1 (or IRS2) would recruit downstream effectors leading to activation of the MAPK cascade and phosphoinositide 3-kinase cascade 24. Phosphatidylinositol 3-kinase-AKT pathway is one of the most important kinase signaling networks in the context of cancer development and treatment 25. Aberrant activation of AKT, the central mediator of this pathway, has been implicated in numerous malignancies including endometrial, hepatocellular and cervical cancer 26, 27, thus regulation and blockage of this kinase is an attractive approach in cancer therapy. However, how to accurately regulate the expression of IRS1, especially via the RNA epigenetic way, subsequently affecting the downstream AKT signaling, still needs to be illustrated.

Herein, we found that RNA reader protein YTHDF2 expression was significantly upregulated in EC samples. YTHDF2 knockdown enhanced the expression of IRS1 and activated AKT signaling pathway, promoted the proliferation and invasion of EC *in vitro*. Together, we identified that the m^6^A reader YTHDF2 as an important regulator of the AKT signaling pathway inhibited the activity of EC, and provided potential targets to prevent the development of EC.

## Methods and materials

### Patient sample collection

All samples were collected under a standard protocol approved by the ethics committee of Shanghai First Maternity and Infant Hospital. The study is compliant with all relevant ethical regulations regarding research involving human participants. All EC clinical samples were diagnosed and assessed in accordance with the International Federation of Gynecology Oncology (FIGO) criteria (2009). The samples must have been diagnosed as endometrial adenocarcinoma and underwent initial surgery. All patients had no history of malignancies. Clinical and pathological data of all patients were retrieved from their records. Patient's characteristics are listed in Table 1. Fresh endometrial tumors and adjacent normal endometrium were separately dissected at the time of surgery and transferred to RNAlater (Thermo Fisher). Tissues were then homogenized in TRIZOL reagent for total RNA isolation or in cell lysis buffer for protein extraction following the manufacturer's instructions.

### Reagents

Antibodies used for experiment were as follows: β-Actin (4970S), Flag (2368S) and HRP-coupled secondary antibodies (7074S) were from CST company. Antibody specific to m^6^A (202003) was from Synaptic Systems. IRS1 (Ab52167) and YTHDF2 (ab220163) antibody were from Abcam (Cambridge, UK). AKT kinase inhibitor (HY-10249A) was from MCE (USA).

### Cell culture and transfection

HEC-1-A, RL95-2 and T-HESCs cell lines were obtained from ATCC and were cultured as previously 13. Cells were transfected with siRNAs (final concentration: 10 nM) or plasmids with Lipofectamine™ 2000 (ThermoFisher, American) according to the manufacturer's instructions. All siRNAs were obtained from GenePharma shown below: IRS1 sense #1: AUACCAAUCAGGUUCUUUGUC, antisense #1: CAAAGAACCUGAUUGGUAUCU, sense #2: AUGAAGAAGAAGUUUUCCGAG, antisense #2: CGGAAAACUUCUUCUUCAUCG; Negative control, Sense: UUCUCCGAACGUGUCACGUTT, Antisense: ACGUGACACGUUCGGAGAATT.

To establish stably transfected HEC-1-A cells, G418 was added (1000 μg/mL) 48 h after transfection and maintained at 500 μg/mL for 3 weeks for positive selection 13. For stably transfected cells, YTHDF2 expression level was confirmed by Western blot. Lentiviruses harboring human YTHDF2, METTL14 or ALKBH5 shRNAs were purchased from OBIO technology (Shanghai, China). HEC-1-A cells were infected with the lentivirus (MOI: 10:1). After HEC-1-A cells were transfected for 72 h, 5 μg/mL puromycin was added for 72 h and maintained at 1 μg/mL for 3 weeks for positive selection of stably knockdown YTHDF2. The sequences encoding the short-hairpin RNA are shown below: Human Ythdf2, #1 AAGGACGTTCCCAATAGCCAA, #2 CAAGGAAACAAAGUACAAAAU; Human Alkbh5, TCGTGTCCGTGTCCTTCTT; Human Mettl14, CGTCAGTATCTTGGGCAAGTT; Negative control, TTCTCCGAACGTGTCACGT.

### RNA extraction and RT-qPCR

Cells were transfected with indicated shRNAs, siRNAs or plasmids. Total RNA was extracted by TRIZOL reagent. RNA was reversed-transcribed using the Reverse Transcription Kit from Toyobo. The reverse transcription products from different samples were amplified by real-time PCR and analyzed as described previously 28. The primer sequences for Q-PCR analysis are listed below: Ythdf2 Forward TAGCCAACTGCGACACATTC, Reverse CACGACCTTGACGTTCCTTT; Irs1 Forward TACATCGCCATCGACGTGAG, Reverse TCAATGCTGGCGTAGGTGTT; Mmp9 Forward GGGACGCAGACATCGTCATC, Reverse TCGTCATCGTCGAAATGGGC; Hprt Forward CCTGGCGTCGTGATTAGTGAT, Reverse AGACGTTCAGTCCTGTCCATAA; Actb Forward CATGTACGTTGCTATCCAGGC, Reverse CTCCTTAATGTCACGCACGAT; Gapdh Forward GCCAAGGTCATCCATGACAACTTTGG, Reverse GCCTGCTTCACCACCTTCTTGATGTC.

### Transwell migration and invasion assay

For invasion and migration assays, we followed the protocol as previously 13. Briefly, cell culture inserts were pre-coated with or without Matrigel (45 μg/insert, Corning), respectively. 40,000 cells per insert were seeded in the upper chamber of the insert and cultured in 300 μL medium supplemented with 1% FBS, and complete medium supplemented with 10% FBS was used in the lower chamber. Following 16-24 h of migration or invasion, calcein-AM (Sigma, 17783) was added to each lower chamber and incubated for 30 mins. Images were collected with a Nikon Eclipse Ti2 with NIS Elements imaging software.

### Cell proliferation and colony formation assay

5000 cells/well were seeded in a 96-well plate. The cell proliferation was assessed by assaying the cells at various time points using the Cell counting kit-8 (Sigma-Aldrich, 96992) 13. For each cell line tested, the signal was normalized to the value observed about 18 h after seeding.

500 cells/well were seeded in 6 well culture dishes. After 7 to 10 days, the cells were washed with PBS, fixed with 4% paraformaldehyde, stained with 0.1% crystal violet (in 25% methanol) 13. Colony numbers were counted manually.

### m^6^A-qRT-PCR

The procedure was adapted from the previous report 29. For m^6^A-qRT-PCR, total RNAs were subjected to mRNA purification by Poly(A) selection (FastTrack mRNA isolation kit, Invitrogen). Following procedure was performed as described in m^6^A-seq assay 30. For comparing the changes of m^6^A peak level, relative enrichment was first normalized with inputs, and then analyzed by comparing the data from m^6^A-immunoprecipitated sample. All samples were analyzed in triplicate qPCR.

### Measurement of RNA lifetime

HEC-1-A cells were seeded in 12 well plates at 80% confluency. After 24 h, Actinomycin D (A4262, Sigma) was added to 5 mg/mL 6 h, 3 h and 0 h before collection 13. The RNA quantities were determined by qRT-PCR. The HPRT1 gene was used as a reference gene when carrying out qPCR.

### Molecular cloning of related genes

Related genes were obtained from human endometrial cell by RT-PCR and subsequently cloned into pcDNA vectors. Each construct was confirmed by sequencing. The corresponding primers used in this study are listed below:

- YTHDF2 Forward CCGGAATTCATGTCGGCCAGCAGCCTCTTG,
- Reverse GCCGGTACCTTATTTCCCACGACCTTGACGTT;
- IRS1 Forward CCGGAATTCATGGCGAGCCCTCCGGAGAGCG,
- Reverse GCCGGTACCCTACTGACGGTCCTCTGGCTGC;
- ALKBH5 Forward CCGGAATTCATGGCGGCCGCCAGCGGCTAC,
- Reverse GCCGGTACCTCAGTGCCGCCGCATCTTCACCTTT.

### Site-directed mutagenesis

m^6^A sites occurred at the consensus RRACH motif (where R indicates G or A; H indicates A, C, or U) 31. According the common or unique m^6^A sites in EC reported previously 8, we mutated these m^6^A sites in IRS1 include: P1-Mut CCAGAAC>GATGC, P2-Mut ACGAGACC>GATC, P3-Mut ACAGAACC>GCAC, P4-Mut CCCAGAC>GAGCT, and P5-Mut TGGAGAC>GTATA, (where > indicates nucleotide substitution). The C within the m^6^A consensus motif was mutated to a G in these sites without changing the encoded amino acid. These mutations were introduced in pGL luciferase plasmids which were generated by site-directed mutagenesis using PCR. We avoided the changes that would alter the RNA secondary structures or codon usage, as predicted by the M-fold and Genscript software 32. All constructs were identified by sequencing.

### Immunoblot

Cells were lysed using Cell Lysis Buffer supplemented with cocktail protease inhibitors and PMSF (Calbiochem). Protein concentrations of the extracts were measured using BCA assays (Pierce, USA) and equalized with the extraction reagent. Equivalent amounts (25 ug) of extract were loaded to SDS-PAGE, and then blotted as described previously 33.

### Statistical analysis

All experiments were independently repeated at least three times. Comparisons between two groups were performed using Student's *t*-test. Data were analyzed with GraphPad Prism software 8.0. Statistical values *p*<0.05 were considered to be statistically significant.

## Results

### YTHDF2 expressed highly in the EC

**Figure 1:**
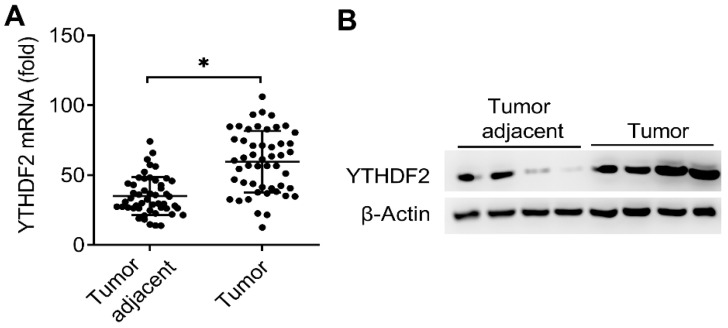
Expression of YTHDF2 significantly upregulated in EC. (**A**) qRT-PCR analysis of YTHDF2 mRNA in EC (Tumor) (n=50) and EC adjacent (Tumor adjacent) (n=50) samples. The gene expression was normalized to that of the β-Actin internal control in each sample. (**B**) Immunoblot analysis of YTHDF2 in EC (Tumor) (n=4) and EC adjacent (Tumor adjacent) (n=4) clinical samples. *p<0.05 (Student's t-test). Data are representative of three independent experiments (mean and s.d. of technical triplicates (**A**).

It had been reported that reduced m^6^A mRNA methylation as an oncogenic mechanism regulated AKT signaling in EC and found that ALKBH5 promoted tumorigenicity of EC via upregulating IGF1R expression 8, 13, we predicted that the m^6^A modification reader proteins maybe also the key regulators of the EC. Interestingly, we found that the mRNA expression of m^6^A reader protein YTHDF2 were significantly upregulated in EC compared to adjacent normal endometrium (50 pairs) (Figure 1A and Table 1). We further confirmed that the protein expression of YTHDF2 were also significantly upregulated in EC (Figure 1B). Taken together, these results indicated that upregulation of YTHDF2 may play an important role in the tumorigenesis of EC, and led us to examine the effects of YTHDF2 on endometrial cell activity.

### YTHDF2 inhibited the activity of endometrial cell

**Figure 2:**
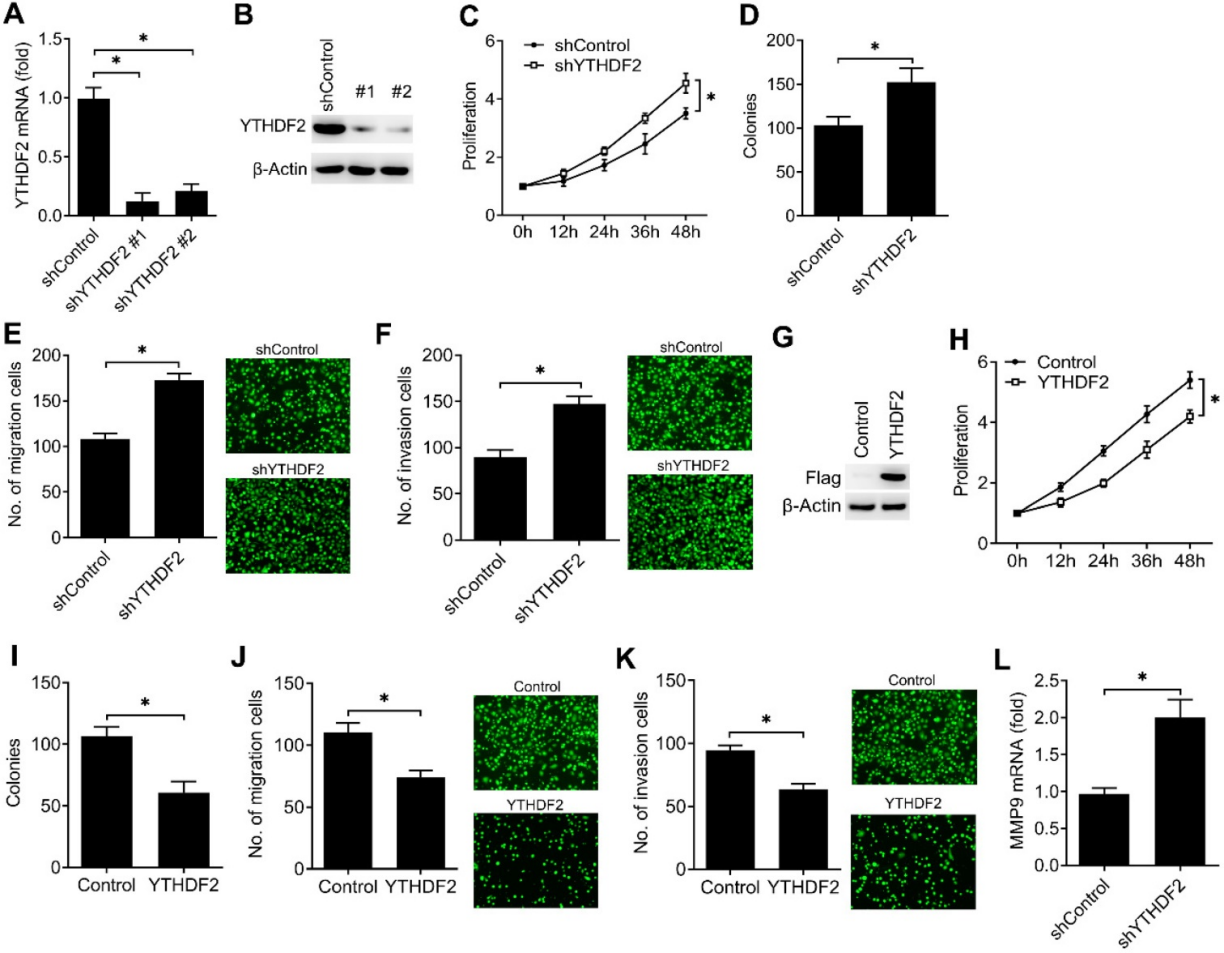
YTHDF2 inhibited the activity of endometrial cell. (**A,B**) qRT-PCR and immunoblot analysis of YTHDF2 in endometrial cell HEC-1-A transfected with lentiviral shControl, YTHDF2 shRNA #1 or #2 as indicated for 72 h. (**C,H**) Cell proliferation measured by CCK8 assay of HEC-1-A cells stably expressing shYTHDF2 (**C**) or YTHDF2 (**H**) as indicated. The value of OD_450_ was normalized to the value about 24 h after cell seeding. (**D,I**) Colony formation analysis of HEC-1-A cells stably expressing shYTHDF2 (**D**) or YTHDF2 (**I**) as indicated. (**E,F**) Migration (**E**) and invasion (**F**) analysis of HEC-1-A cells stably expressing shControl or shYTHDF2 by Transwell assay. The numbers of migration and invasion cells were counted by ImageJ software. (**G**) Immunoblot analysis of HEC-1-A cells stably expressing YTHDF2 as indicated. (**J,K**) Migration (**J**) and invasion (**K**) analysis of HEC-1-A cells transfected with YTHDF2 plasmid by Transwell assay. (**L**) qRT-PCR analysis of MMP9 mRNA in HEC-1-A stably expressing shControl and shYTHDF2, and stimulated with IGF for 6 h. *p<0.05 (Student's t-test). Data are representative of three independent experiments (mean and s.d. of technical triplicates (**A**,**C-F**,**H-L**).

To investigate the biological functions of YTHDF2, we examined the effects of YTHDF2 knockdown or overexpression on endometrial cell activity. Firstly, we confirmed that YTHDF2 was significantly knocked down by using two different lentiviral shRNA sequences (Figure 2A, B). We found that YTHDF2 knockdown significantly enhanced the proliferation, migration and invasion of endometrial cells (Figure 2C-F). Furtherly, overexpression of YTHDF2 significantly inhibited the proliferation, migration and invasion of endometrial cells (Figure 2G-K), suggesting that YTHDF2 could inhibit the activity of endometrial cells.

To further determine the downstream signal pathway of YTHDF2 in regulating the activity of endometrial cell, we confirmed that knockdown of YTHDF2 enhanced the MMP9 expression of endometrial cells (Figure 2L). These results indicated that YTHDF2 inhibited the activity of endometrial cell probably via inhibiting MMP9 expression.

### YTHDF2 inhibited the activity of other endometrial cell lines

**Figure 3:**
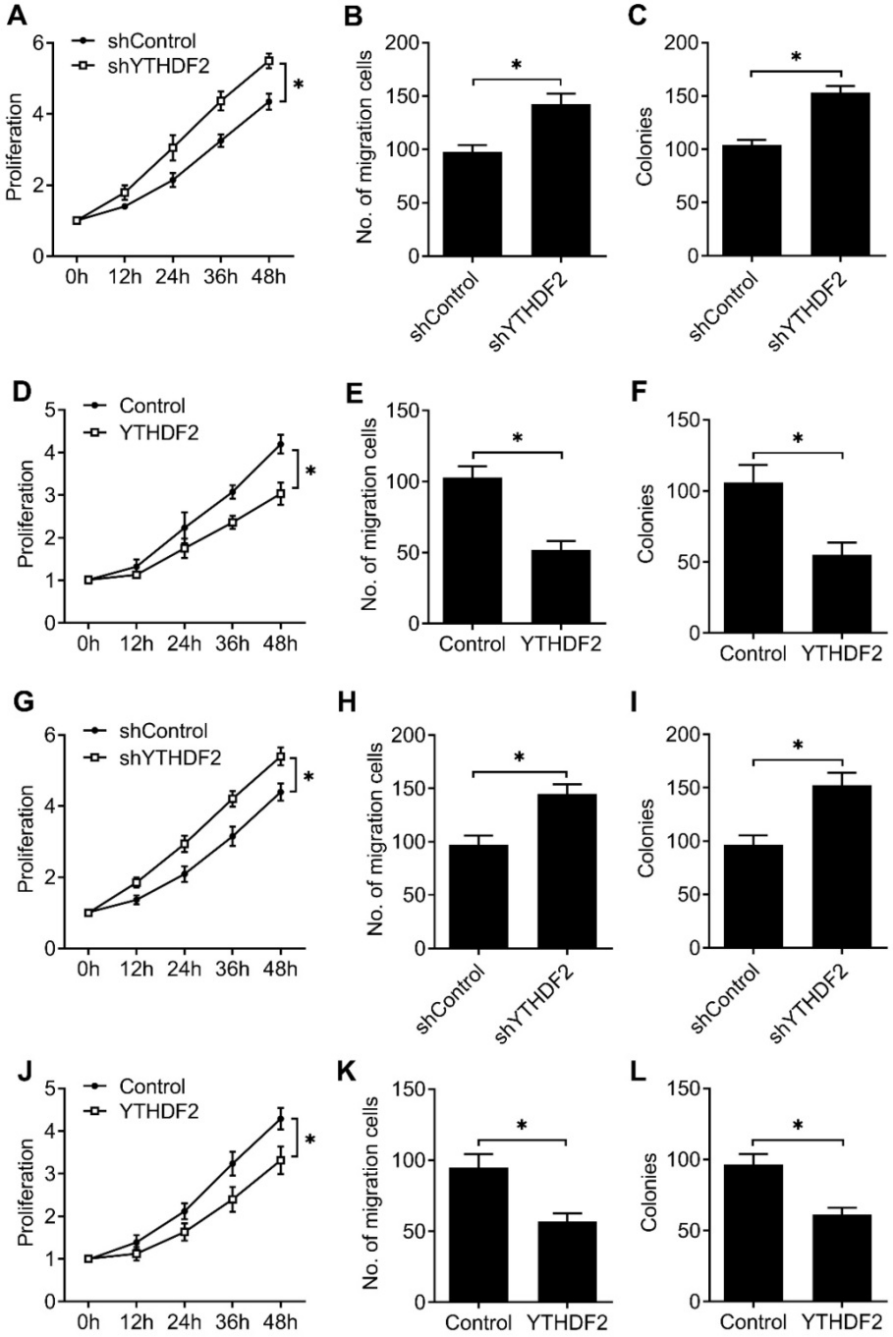
** YTHDF2 inhibited the activity of endometrial cell RL95-2 and normal non-transformed endometrial cells (T-HESCs).** (**A,D,G,J**) Cell proliferation measured by CCK8 assay of endometrial cell RL95-2 (**A,D**) and T-HESCs (**G,J**) transfected with lentiviral shYTHDF2 or YTHDF2 plasmid as indicated. (**B,E,H,K**) Migration analysis of endometrial cell RL95-2 (**B,E**) and T-HESCs (**H,K**) transfected with lentiviral shYTHDF2 or YTHDF2 plasmid as indicated by Transwell assay. The numbers of migration cell were counted by ImageJ software. (**E,F,I,L**) Colony formation analysis of endometrial cell RL95-2 (**E,F**) and T-HESCs (**I,L**) transfected with lentiviral shYTHDF2 or YTHDF2 plasmid as indicated. *p<0.05 (Student's t-test). Data are representative of three independent experiments (mean and s.d. of technical triplicates (**A-L**).

To furtherly confirm that our results extend beyond the HEC-1-A EC cell line, we tested the effects of knockdown and overexpression of YTHDF2 in EC cell line (RL95-2), as well as normal non-transformed human endometrial stromal cells (T-HESCs). We found that YTHDF2 knockdown significantly enhanced the proliferation, migration and invasion of RL95-2 (Figure 3A-C) and T-HESCs (Figure 3G-I) cell lines, while overexpression of YTHDF2 significantly inhibited the proliferation, migration and invasion of RL95-2 (Figure 3D-F) and T-HESCs (Figure 3J-L) cell lines, these results furtherly proved that YTHDF2 could inhibit the activity of endometrial cell.

### YTHDF2 bind m^6^A methylation of IRS1 transcripts in EC

**Figure 4:**
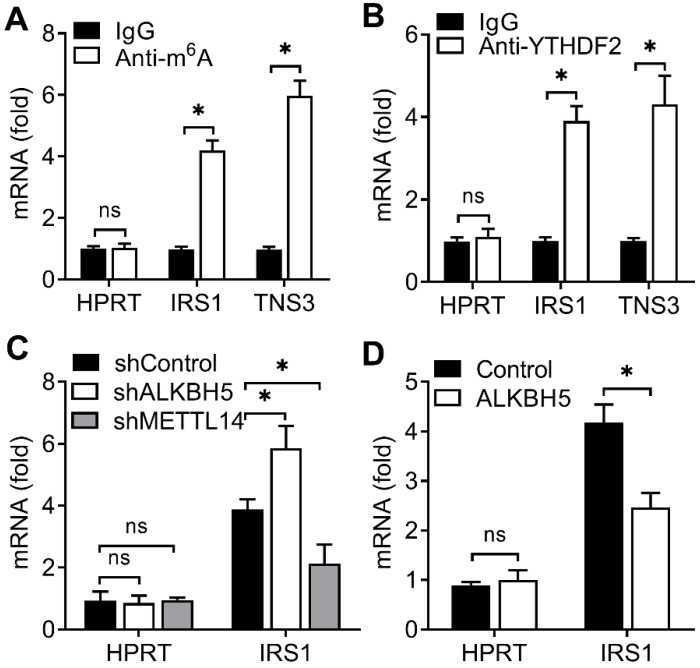
YTHDF2 bind to the m^6^A methylation of IRS1 mRNA. (**A**,**B**) Enrichment fold of the indicated mRNA transcripts in m^6^A IP (**A**), or YTHDF2 IP (**B**) versus mRNA input control. (**C**,**D**) Enrichment fold of the indicated mRNA transcripts in m^6^A IP versus mRNA input control in HEC-1-A after transfected with the indicated shRNA for 72 h (**C**) or plasmids (**D**) for 48 h. Each transcript was quantified by qRT-PCR. ns: not significant; *p<0.05 (Student's t-test). Data are representative of three independent experiments (mean and s.d. of technical triplicates (**A,B**).

Due to that YTHDF2 inhibited endometrial cell activity and YTHDF2 as an important m^6^A reader protein, we intended to identify the key substrate transcripts with methylation in EC. Previous m^6^A-seq map showing m^6^A methylation transcripts were enriched in KEGG terms related to RTK-AKT signaling pathway 8. We hypothesized that YTHDF2 might inhibit the activity of endometrial cell through inhibition the downstream of the RTK (IGF) signaling pathway. Intriguingly, we found that IRS1 had m^6^A methylation modifications in EC according to m^6^A-seq data 8. We performed immunoprecipitation of the HEC-1-A mRNA, and found that, in addition to negative control HPRT, IRS1 and TNS3 (positive control) mRNAs were enriched in the m^6^A and YTHDF2 antibody-bound fraction (Figure 4A,B), confirming the presence of m^6^A sites on the IRS1 transcripts and YTHDF2 can bind to IRS1 transcripts, so we selected IRS1 to be studied.

Next we intend to investigate whether ALKBH5 targets the m^6^A modification of IRS1 mRNA. We performed immunoprecipitation of the HEC-1-A mRNA, and found that, IRS1 showed increased enrichment in the m^6^A antibody-bound fraction when ALKBH5 was knocked down, but decreased enrichment when the m^6^A 'writer' METTL14 was knocked down (Figure 4C), and the enrichment of IRS1 mRNAs to m^6^A antibody decreased in HEC-1-A after overexpression of ALKBH5 (Figure 4D). The above results revealed that YTHDF2 regulated the activity of endometrial cell probably via binding the IRS1 transcripts, and m^6^A modification in IRS1 transcripts can be erased by ALKBH5 and added by METTL14.

### YTHDF2 promoted the degradation of IRS1 mRNA and inhibited IRS1 expression

**Figure 5:**
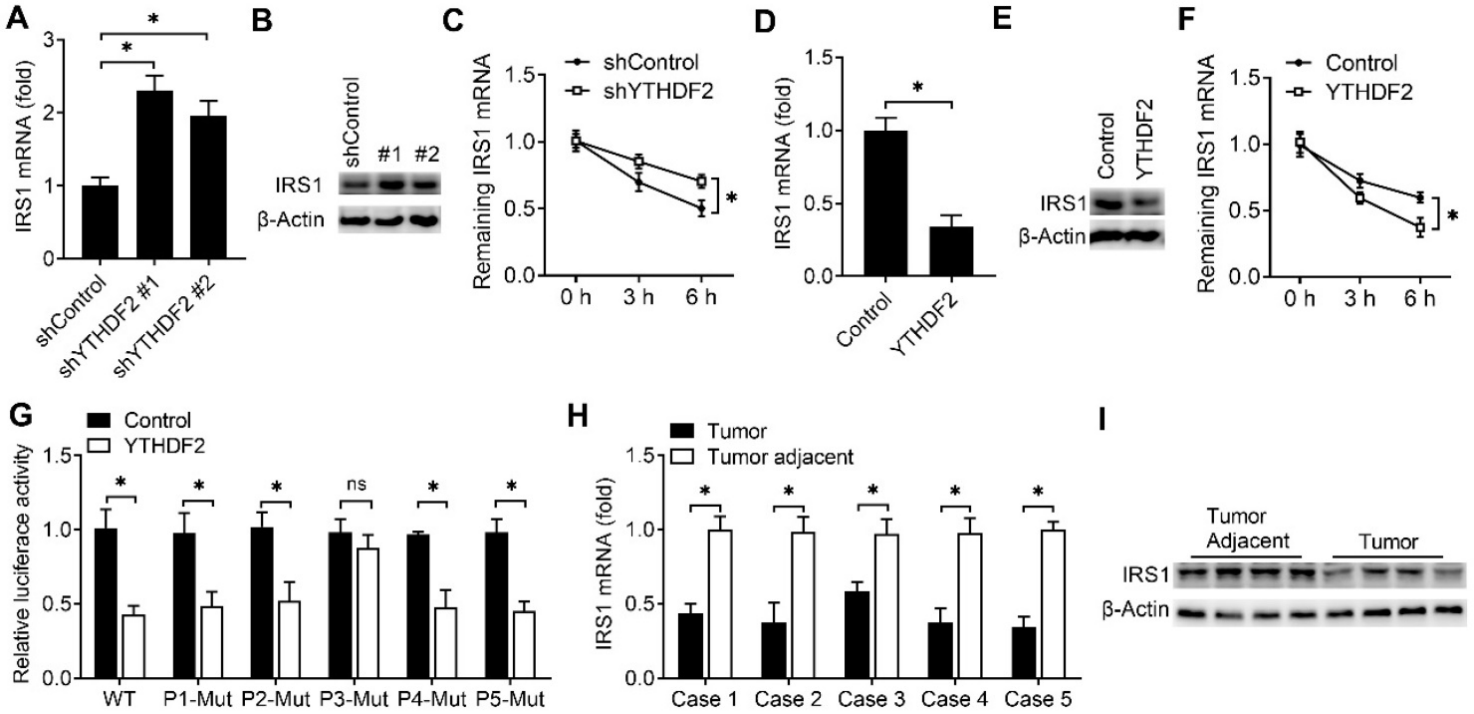
YTHDF2 promoted IRS1 mRNA degradation and inhibited IRS1 expression. (**A**,**B,D,E**) qRT-PCR and immunoblot analysis of the IRS1 mRNA and protein in HEC-1-A transfected with shYTHDF2 for 72 h (**A,B**) or plasmids as indicated for 48 h (**D,E**). (**C**,**F**) RNA lifetime for IRS1 in HEC-1-A cells transfected with shYTHDF2 (**C**) or plasmids (**F**) was determined by monitoring transcript abundance after transcription inhibition (TI). (**G**) Luciferase activity analysis in HEC-1-A cells after co-transfection with YTHDF2 and IRS1 m^6^A site mutants as indicated. (**H**) qRT-PCR analysis of IRS1 mRNA expression in EC from five cases. (**I**) Immunoblot analysis of IRS1 in EC (Tumor) (n=4) and EC adjacent (Tumor adjacent) (n=4) clinical samples. ns: not significant; *p<0.05 (Student's t-test). Data are representative of three independent experiments (mean and s.d. of technical triplicates (**A**-**C**,**E**,**F**,**H**).

We next explored that whether the expression of IRS1 were affected after binding with YTHDF2. We found that knockdown of YTHDF2 increased the mRNA and protein levels of the IRS1 transcripts and IRS1 transcripts showed decreased RNA decay rates upon knockdown of *YTHDF2* (Figure 5A-C). Overexpression of YTHDF2 decreased the mRNA and protein levels of the *IRS1* transcripts and IRS1 transcripts showed increased RNA decay rates upon YTHDF2 overexpression (Figure 5D-F). Next, we intend to explore how the YTHDF2 affect the IRS1 mRNA decay. By analyzing the m^6^A methylated RNA immunoprecipitation sequencing (meRIP-seq) data available for EC tissue and cell lines 8, we found that there are 5 conserved putative m^6^A sites in the coding region of IRS1 mRNA (P1-P5). We mutated these m^6^A sites without changing the amino acids they encode accordingly and generated five mutant plasmids lacking m^6^A sites in the IRS1 mRNA (P1-Mut, P2-Mut, P3-Mut, P4-Mut and P5-Mut). We found that overexpression of YTHDF2 reduced luciferase activity of WT m^6^A motif IRS1 (P1-5) and m^6^A motif mutant IRS1 (P1-Mut, P2-Mut, P4-Mut and P5-Mut) reporters, but not that of the P3 m^6^A motif mutant IRS1 (P3-Mut) reporter (Figure 5G), suggesting that m^6^A modification of IRS1 mRNA at P3 is critical for IRS1 mRNA stability.

To investigate whether the expression of IRS1 protein in EC adjacent and EC were consistent with the YTHDF2, we found that the mRNA and protein level of IRS1 were indeed downregulated in EC when compared with adjacent normal endometrium (Figure 5H,I). These results indicated that YTHDF2 inhibited the expression of IRS1 via promoting the decay of IRS1 transcripts in endometrial cells.

### YTHDF2 regulated the activity of endometrial cell via targeting IRS1

**Figure 6:**
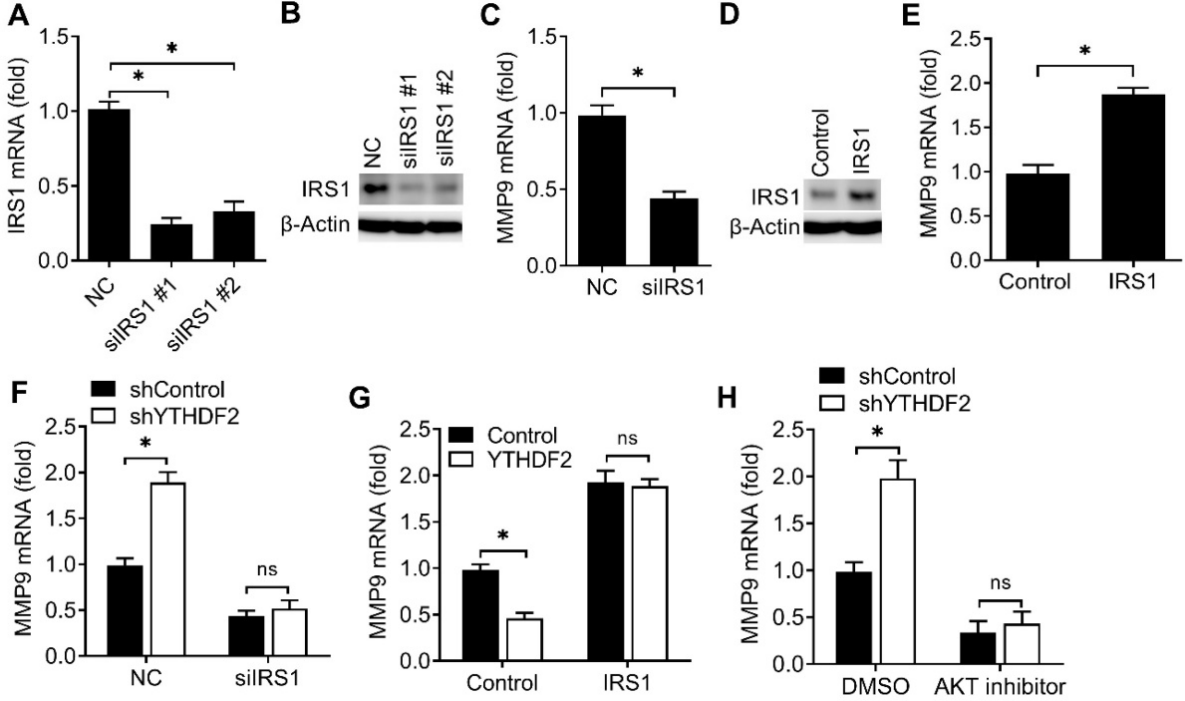
YTHDF2 regulated the activity of endometrial cell via targeting IRS1. (**A**,**B**) qRT-PCR and immunoblot analysis of IRS1 in HEC-1-A transfected with negative control (NC), IRS1 siRNA #1 or #2 as indicated for 48 h. (**C**,**E-H**) qRT-PCR analysis of MMP9 mRNA in HEC-1-A transfected with siRNA (**C,F**), plasmids (**E,G**) or treated with AKT inhibitor (**H**) as indicated, and stimulated with IGF for 6 h. (**D**) Immunoblot analysis of IRS1 in HEC-1-A transfected with the IRS1 plasmids for 48 h. ns: not significant; *p<0.05 (Student's t-test). Data are representative of three independent experiments (mean and s.d. of technical triplicates (**A,C,E-H**).

We then investigated the potential roles of IRS1 in regulating the activity of endometrial cell. Firstly, we confirmed that IRS1 was efficiently knocked down by two different siRNAs (Figure 6A,B). We found that knockdown of IRS1 significantly inhibited the MMP9 expression of endometrial cells (Figure 6C), and overexpression of IRS1 significantly promote the MMP9 expression of endometrial cell (Figure 6D,E), which was a phenocopy of our results obtained from YTHDF2.

We next asked whether YTHDF2 regulated the activity of endometrial cell mainly through IRS1 pathway. We found that YTHDF2 knockdown-mediated promotion of MMP9 production was rescued by IRS1 knockdown (Figure 6F), suggesting that IRS1 knockdown counteracted the effect of YTHDF2 knockdown. Accordingly, overexpression of IRS1 reversed the YTHDF2 overexpression-mediated inhibition of MMP9 production (Figure 6G), thus determining that the role of YTHDF2 in inhibiting endometrial cell MMP9 production was dependent on IRS1. Furtherly, YTHDF2 knockdown-mediated promotion of MMP9 production was blocked by AKT kinase inhibitor (Figure 6H). Taken together, we found that YTHDF2 regulated the activity of endometrial cell mainly via targeting IRS1.

On the basis of our findings, we propose the following working model to explain how YTHDF2 inhibit the activity of endometrial cell. YTHDF2 expression significantly upregulates in EC, and YTHDF2 bind to the P3 m^6^A modifications of IRS1 transcripts and inhibit IRS1 expression, which lead to the inhibition of the AKT/MMP9 signaling pathway, subsequently decreases the invasion and migration of endometrial cells.

## Discussion

We find that the m^6^A reader protein YTHDF2 expression upregulated in EC. YTHDF2 can inhibit the proliferation and invasion of endometrial cell *in vitro*. Mechanically, YTHDF2 inhibit the expression of IRS1 via promoting IRS1 mRNA decay and decrease the expression of MMP9, which can inhibit the invasion of endometrial cell. Together, our results indicate that YTHDF2 may be a potential target to prevent the tumorigenicity of EC.

It was reported that YTHDF2 highly expressed in many cancers and played important functions in various cancers 34, 35. We also found that YTHDF2 expression was upregulated in EC and overexpression of YTHDF2 can inhibit the proliferation and invasion of endometrial cell line, this indicated that the functions of YTHDF2 depend on the specific type of cancer and the specific function *in vivo* need to be investigated in the future. Meanwhile, we intend to explore whether the expression of YTHDF2 or IRS1 associated with patient survival. Using the TCGA databases to analyze the expression and survival of YTHDF2 or IRS1 in uterine corpus endometrial carcinoma (UCEC), we found that the YTHDF2 was significantly upregulated and IRS1 was significantly downregulated in primary tumor compared to normal tissue, this is consistent with our findings, but the effect of YTHDF2 or IRS1 expression level on UCEC patient survival is not significantly (data not shown), this results beyond our expected results, and the association between YTHDF2 and the survival of UCEC need to be explored in the future.

The dynamically m^6^A methylated mRNA could have effects on cellular physiology, especially if the key transcripts are affected 36. It is reported that reduced m^6^A methylation of mTOR and PHLPP2 could promote EC cell proliferation by affecting the AKT signaling pathway 8 and m^6^A demethylation of IGF1R could also promote the activity of EC cell 13. We also found that overexpression of YTHDF2 inhibited the activity of endometrial cell by binding the m^6^A modification of IRS1 and decreasing IRS1 expression. These results indicated that the activity of endometrial cell was regulated by many key proteins in AKT signaling pathway, and these proteins keep the dynamic equilibrium of endometrial cell activity. Furtherly, we found that the IRS1 have five potential m^6^A site, but only P3 site is critical for the YTHDF2 mediated IRS1 decay, this means that the conserved base sequence and advanced structure of m^6^A site is very important for the specific reader to recognize, maybe other m^6^A sites were recognized by other m^6^A readers and play other functions, this need to be studied in the future.

Previously reported that IGF signaling pathway played key roles in the regulation of normal uterine physiology and the development of endometrial hyperplasia 37, and the IRS1 is a key mediator in oncogenic insulin-like growth factor signaling 38, 39. IRS1 has been identified to be highly expressed in breast cancer, and regulates the sensitivity of breast cancer cells to chemotherapy 38. We found that YTHDF2 could regulate the expression of IRS1 via m^6^A RNA epigenetic way, and IRS1 significantly downregulated in EC compared to EC adjacent, this is consistent with the upregulation of YTHDF2 in EC. We speculated that decreased IRS1 expression is probably one of the main mediators of decreased activity in cells with YTHDF2 overexpression, as overexpression of IRS1 is sufficient to rescue the inhibition of the MMP9 expression mediated by YTHDF2 overexpression. These findings may be applicable beyond endometrial cell invasion to other cancers driven by YTHDF2 overexpression. However, we cannot rule out the involvement of other signaling pathways that could be altered directly or indirectly by YTHDF2 expression, this need to be further investigated.

## Figures and Tables

**Table 1:** Tissue characteristics of patient with EC

Characteristics	Numbers	Percent (%)
**Age (years)**		
31-40	2	4.0
41-50	8	16.0
51-60	25	50.0
61-70	14	28.0
71-80	1	2.0
**FIGO 2009 Staging**		
I	37	74.0
II	9	18.0
III	3	6.0
IV	1	2.0
**Grade**		
G1	28	56.0
G2	15	30.0
G3	7	14.0
